# Constant Optical Power Operation of an Ultraviolet LED Controlled by a Smartphone

**DOI:** 10.3390/s21144707

**Published:** 2021-07-09

**Authors:** Ching-Hua Chen, Jia-Jun Zhang, Chang-Han Wang, Yu-Chia Chang, Pinghui S. Yeh

**Affiliations:** 1 Department of Electronic and Computer Engineering, National Taiwan University of Science and Technology, Taipei 106, Taiwan; M10702303@mail.ntust.edu.tw (C.-H.C.); M10702321@mail.ntust.edu.tw (J.-J.Z.); M10702336@mail.ntust.edu.tw (C.-H.W.); M10802334@mail.ntust.edu.tw (Y.-C.C.); 2 Graduate Institute of Electro-Optical Engineering, National Taiwan University of Science and Technology, Taipei 106, Taiwan

**Keywords:** UV sensors, light-emitting diodes, monitoring photodiodes, smartphone, App, Arduino

## Abstract

Constant light power operation of an ultraviolet (UV) LED based on portable low-cost instrumentation and a monolithically integrated monitoring photodiode (MPD) has been reported for the first time. UV light irradiation has become one of the essential measures for disinfection and sterilization. Monitoring and maintaining a specified light power level is important to meet the criteria of sterilization. We built a module composed of a monolithically integrated UV LED and MPD, a transimpedance amplifier, an Arduino Uno card, a digital-to-analog converter and a Bluetooth transceiver. An Android App that we wrote remotely controlled the UV LED module via Bluetooth. The Arduino Uno card was programmed to receive demands from the smartphone, sent a driving voltage to the LED and returned the present MPD voltage to the smartphone. A feedback loop was used to adjust the LED voltage for maintaining a constant light output. We successfully demonstrated the functioning of remote control of the App, and the resultant UV LED measured power remained the same as the setting power. This setup can also be applied to visible or white LEDs for controlling/maintaining mixed light’s chromaticity coordinates or color temperature. With such controlling and internet capability, custom profiling and maintenance of precision lighting remotely would be possible.

## 1. Introduction

Ultraviolet (UV) light-emitting diodes (LEDs) are used in many applications, such as epoxy curing, air/water sterilization, surface disinfection and biomedical assays. UV-C light can kill viruses or impede their proliferation. However, the effectiveness of these applications requires a minimum UV light intensity for a certain amount of time; thus, real-time monitoring of the light power level is essential. Typically, AlInGaN-based UV LEDs have a shorter lifetime than visible LEDs [1,2,3]. Currently, constant current operation is used when evaluating the degradation behavior of UV LEDs [1,2,3]. For monitoring the output power of an edge-emitting laser diode or super luminescent diode, positioning an external Si or InGaAs photodiode at the rear end of the irradiating device is often used. To monitor surface-emitting LEDs (including UV LEDs), adding an external Si or wide-bandgap photodiode would sacrifice some useful light power as well as compactness. Moreover, an external photodetector would be sensitive to ambient lighting and its monitoring responsivity would change with the relative distance and angle between the LED and photodetector, which is difficult to maintain. On the other hand, on-chip power detection was possible by using the same active layer for both emission and detection under forward and reverse biases, respectively. Nevertheless, the responsivity of the photodiode was fairly low for a basic p–i–n photodiode because the emission wavelength of the LED was near the cutoff wavelength of the photodiode. Tchernycheva et al. reported the integration of GaN-based single-wire LEDs and photodetectors optically coupled with silicon nitride waveguides [4]. To achieve good spectral matching between the emission wavelength and the detection range, different active regions containing five narrow InGaN/GaN quantum wells and one thick InGaN layer were employed for the LED and photodetector, respectively.

Several research groups reported the monolithic integration of LEDs and photodetectors of different configurations for optical communication or interconnection. For example, Jiang et al. reported the integration of blue LEDs and UV Schottky barrier photodiodes as transmitters and receivers, respectively [5]; Wang et al. demonstrated in-plane data transmission between LEDs, waveguides and photodiodes [6,7,8]. Li et al. fabricated integrated photodiodes, LEDs and waveguides, selectively detached from a substrate for reducing crosstalk [9,10]. Liu et al. reported the monolithic integration of an LED, a high-electron mobility transistor and dual-wavelength photodiodes through selective area epitaxy on a sapphire substrate [11]. Li et al. demonstrated a visible light communication system using a micro-LED display as the transmitter and a smartphone camera as the receiver [12]. Lyu et al. reported the monolithic integration of UV LEDs and photodetectors on a p-GaN/AlGaN/GaN/Si platform [13]. For optogenetics, Sekiguchi et al. developed an optical probe using a monolithically integrated 460 nm micro-LED, photodiode and polymer waveguide [14]. To detect ambient UV light sensitively, Yeh et al. fabricated integrated phototransistors and LEDs based on an LED epitaxial wafer [15]. Chiu et al. used a visible LED to display a warning light while detecting ambient UV light with an on-chip photodiode [16]. Nevertheless, none of the above-mentioned photodetectors were used for monitoring the surface-emitting power output of the integrated LEDs.

In the year 2020, we reported the on-chip power monitoring of UV LEDs utilizing a sapphire substrate as a slab waveguide, which coupled part of the emission power towards a monitoring photodiode (MPD) shaped in a loop surrounding the UV LED [17,18]. The top of the MPD was completely covered by metal to block any ambient incoming light. A transimpedance amplifier (TIA) was employed to increase its responsivity. Recently, Yin et al. reported monolithically integrated photodiodes in white LEDs to monitor the fluctuation of light intensity over time [19]. They studied the effect of various MPD locations on the light intensity distribution of the LED and concluded that a central location was the best. These aforementioned studies performed real-time monitoring of the surface-emitting power of LEDs. However, there was no automatic mechanism provided to maintain the power level. In this work, based on a self-made integrated LED/MPD device, we further built a module or subsystem (including programming) that consisted of an Arduino Uno card, a digital-to-analog converter (DAC) to control the LED/MPD and a Bluetooth transceiver to remotely communicate the input/output with a smartphone. The networking capability of a smartphone enables internet of things. Ishigaki et al. had ever developed a mobile radiation monitoring system in Fukushima, Japan, utilizing a combination of a discrete p–i–n photodiode connected to a smartphone [20]. Using an App that we wrote, we were able to simply select the desired UV light power and read the measured light power. Constant optical power operation of an UV LED remotely was achieved.

The following report is organized into five sections. The layout and design of the whole module are presented in Section 2. The Arduino and App programming are described in Section 3 and Section 4, respectively. Implemented results and discussion are presented in Section 5. Conclusions are given in Section 6.

## 2. Module Design

**Figure 1:**
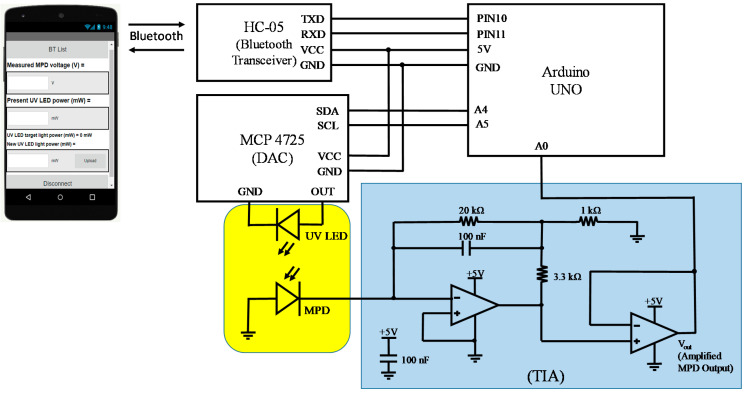
Layout of the module communicating with a smartphone, where DAC, MPD and TIA represent digital-to-analog converter, monitoring photodiode and transimpedance amplifier, respectively.

**Figure 2:**
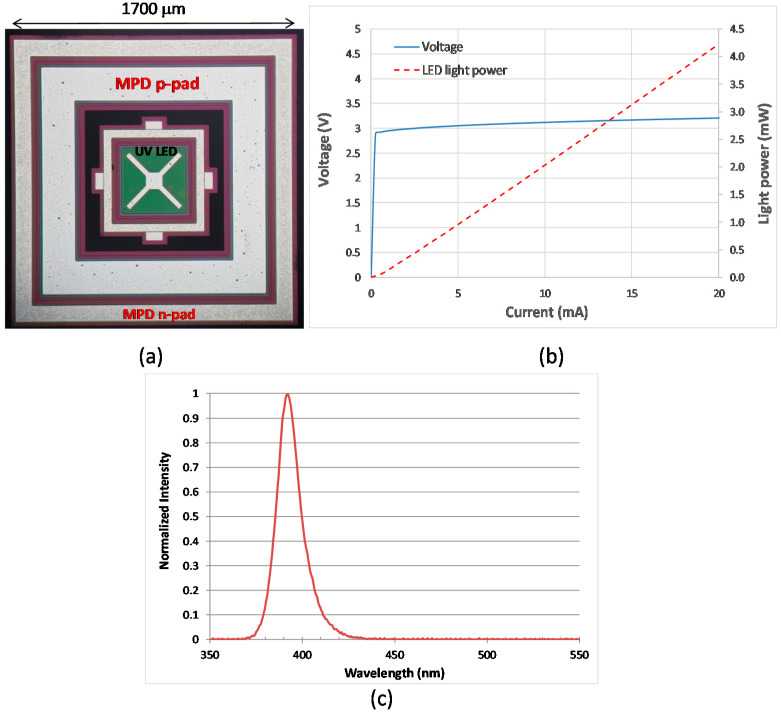
(**a**) A photo of the monolithically integrated UV LED and MPD; (**b**) V–I–L characteristics of the UV LED; (**c**) Emission spectrum of the UV LED.

Figure 1 shows the layout of the module communicating with a smartphone via Bluetooth. The module consisted of a monolithically integrated UV LED and MPD, a TIA, an Arduino Uno card, a DAC (Microchip MCP 4725) and a wireless Bluetooth module (HC-05). The Arduino Uno is an open-source microcontroller board based on the microchip ATmega328P [21]. The key technical specifications of the Arduino Uno and Bluetooth module HC-05 are listed in Table 1 and Table 2, respectively [21,22]. The Arduino card was the central controller that received demands from the smartphone, converted a required LED light power to a driving voltage applied to the LED and received the resultant MPD voltage from the TIA. The baud rate was 9600 bit/s. When there was no demand from the smartphone, the Arduino Uno continuously adjusted the LED driving voltage for attaining/maintaining the required light power level, and transmitted the present MPD voltage to the smartphone. The monolithically integrated UV LED and MPD, as shown in Figure 2a, were fabricated in the same manner as described in our previous report [17]. The UV LED was surrounded by a p–i–n photodiode shaped in a loop. The LED downward-emitting light towards the sapphire substrate was partially scattered and collected by the MPD that was linearly proportional to the surface-emitting power [17]. The MPD was completely covered with electrode metal on top to block any external light and thus detect internal incoming light only. The monitoring responsivity per LED surface-emitting power of the device used in this report was approximately 7.4 mA/W at zero bias voltage. The transimpedance gain of the TIA was approximately 8.9 × 10^4^ V/A, resulting in a monitoring responsivity of approximately 0.66 V/mW at zero bias voltage. The voltage–current–light (V–I–L) characteristics of the UV LED are plotted in Figure 2b. The emission spectrum had a peak wavelength and a full width at half maximum of approximately 392 and 15 nm, respectively.

## 3. Arduino Programming

**Figure 3:**
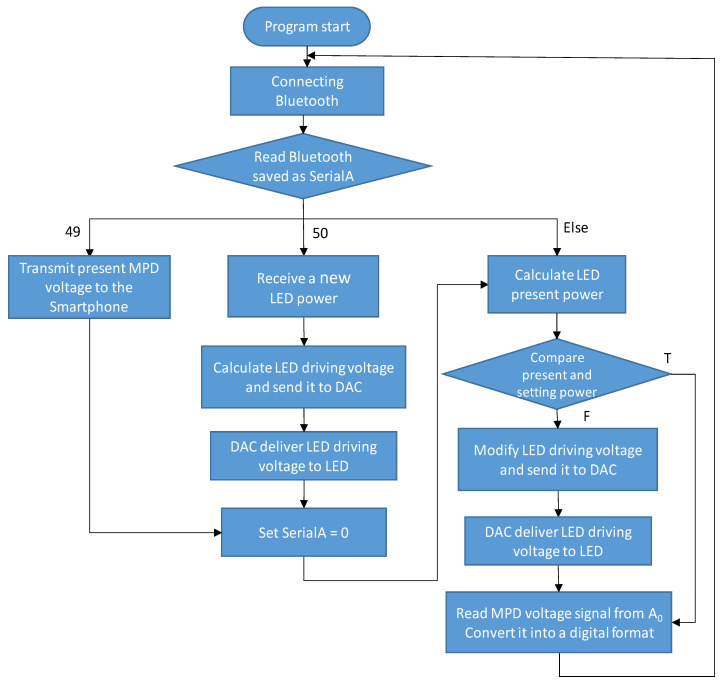
Flow chart of the Arduino program for the UV LED module.

Figure 3 illustrates the flow chart of the Arduino programming. First, the Arduino Uno card read the input parameter (named SerialA) from the Bluetooth transceiver. When this number was 50, indicating that the smartphone was uploading a new LED light power, Arduino read the new power, calculated the associated LED driving voltage based on the voltage–light relationship saved in the program and sent the voltage to DAC. Next, the DAC converted the voltage from a digital number into an analog signal, which was then applied to the UV LED. Consequently, the MPD detected the light power, resulting in an amplified MPD voltage signal delivered back to the pin **A_0_** of Arduino. The parameter SerialA was then set to be zero. Secondly, Arduino calculated the present LED light power according to the measured MPD voltage and the corresponding monitoring responsivity saved in the program (Note that we assumed that the monitoring responsivity did not change over time or at a rate significantly slower than the degradation rate of the UV LED). When the present LED light power was lower (or higher) than the setting power, the LED driving voltage would be increased (or reduced) correspondingly. This function was performed by a program loop that compared the measured power and setting power; when the difference was smaller than a given number, the loop ended; otherwise, the program calculated an estimated increase or decrease in LED driving voltage to deploy, waited some time (allowing Arduino to check if any new demands coming from the smartphone) and then re-compared the two powers (Note that when Arduino calculated the present LED power, an updated MPD voltage from pin A_0_ was used). On the other hand, when the received number via Bluetooth was 49, indicating that the smartphone wanted to read the present MPD voltage, Arduino would transmit it to the smartphone and reset SerialA to be zero. Then, it continuously monitored and maintained the present LED light power to be the same as the setting power.

## 4. Android App Programming

**Figure 4:**
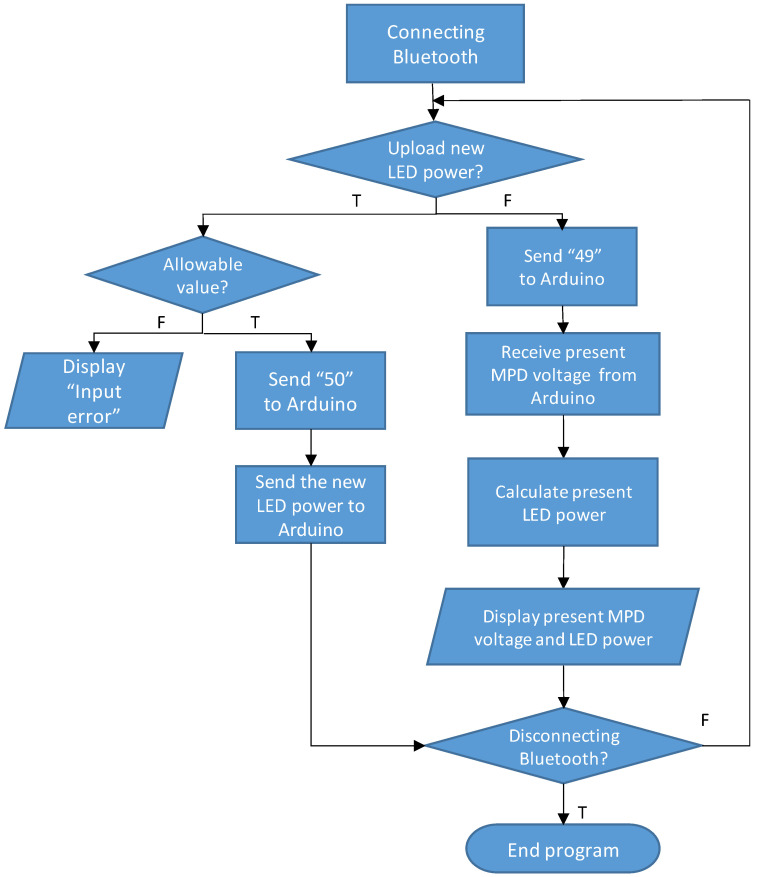
Flow chart of the App program to control the UV LED module.

We wrote an Android App program based on MIT App Inventor 2 [23], which is a free open-source software program to create Apps for two operating systems, Android and iOS, using a graphical user interface. The flow chart of our program is shown in Figure 4. The App program started with connecting to the Bluetooth transceiver of the UV LED module. When the user did not choose to upload a new LED light power, the program set a value of 49 to the parameter SerialA and read the present MPD voltage from Arduino. The present LED light power was then calculated based on the monitoring responsivity saved in the program, which was the same as the one saved in the Arduino program. Hence, both the measured MPD voltage and present LED light power were displayed on the screen of the smartphone. When the user chose to upload a new LED light power, the program first checked whether the power was within the allowable range that set a minimum required power, associated with a target light intensity, and a maximum compliance power to protect the LED given that it had not been packaged. Once the new LED power was allowed, the App program set a value of 50 to the parameter SerialA and sent the new LED power to Arduino.

## 5. Results and Discussion

**Figure 5:**
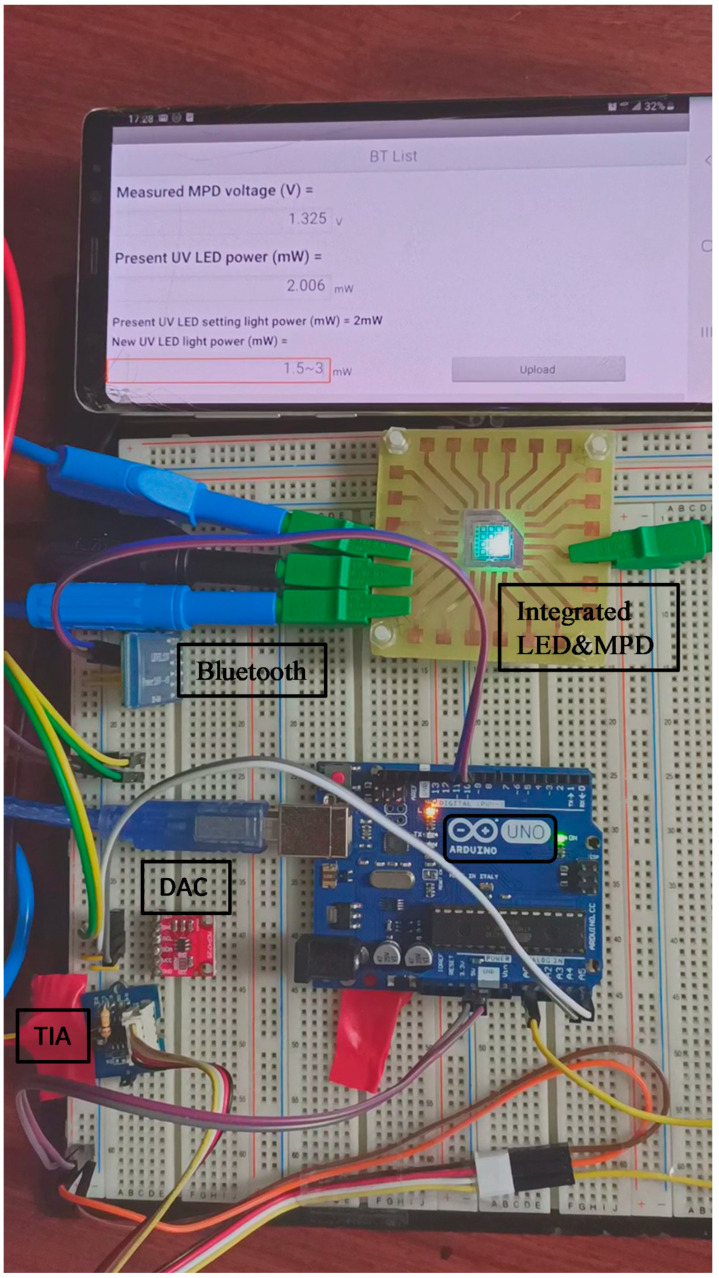
A photo of the UV LED module under constant optical power operation, where MPD, DAC and TIA represent monitoring photodiode, digital-to-analog converter and transimpedance amplifier, respectively.

Figure 5 illustrates the operation of the UV LED module built on a breadboard. Each component is marked in black. The experiment was conducted under general room lighting. (On-chip power monitoring was insensitive to ambient lighting.) The smartphone’s screen showed a measured MPD voltage of 1.32 V and a present UV LED light power of 2.0 mW, when the setting light power was 2.0 mW. We could remove the smartphone and leave the UV LED on at the setting power level continuously under constant light power operation. The bottom line on the screen was the place to upload a new UV LED light power, which was restricted in the range of 1.5–3.0 mW in this demonstration, because the UV LED was mounted p-side up, having a sapphire substrate of poor thermal conductivity. To operate at a higher power level, we may (1) scale up the area size of the integrated UV LED/MPD device; (2) combine multiple devices in parallel or (3) package the device for good heat dissipation. Moreover, although the emission of this device was UV-A light, the same principle and instrumentation can be applied to UV-B and UV-C LEDs. Hence, a reliable UV lighting system can be constructed and some applications can be implemented, e.g., UV-B dose monitoring to meet individual daily UV-B dose [24]. This instrumentation can also be applied to GaN-based visible or white LEDs for controlling/maintaining mixed light’s chromaticity coordinates or color temperature. Furthermore, with such controlling and internet capability, custom profiling and maintenance of lighting remotely would be possible.

**Figure 6:**
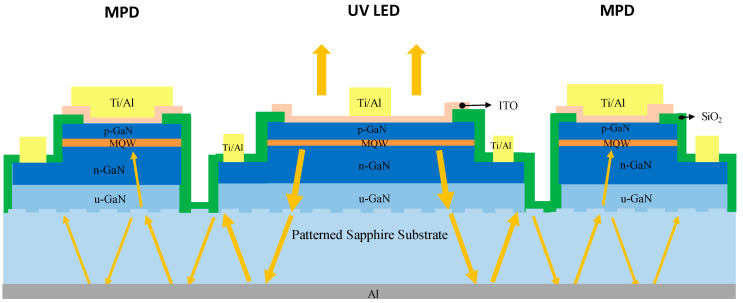
Schematic diagram of the integrated UV LED and MPD, with yellow arrows illustrating the propagation of some light rays as examples, where ITO represents indium tin oxide.

**Figure 7:**
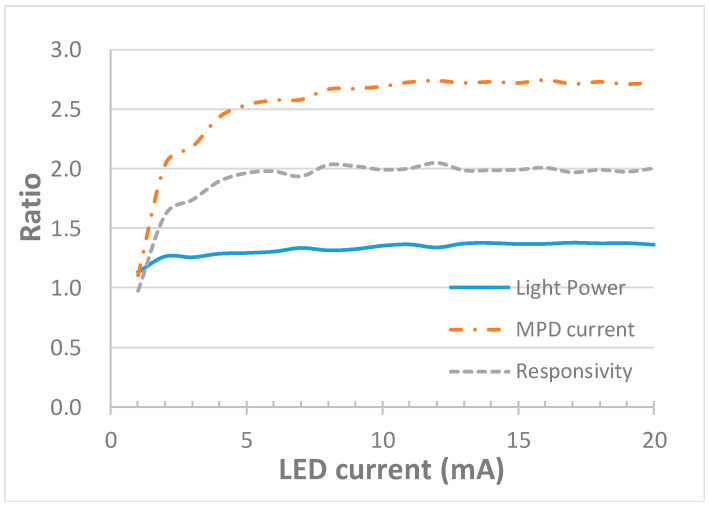
The ratio of after to before Al deposition, in terms of UV LED light power, MPD current at zero bias and monitoring responsivity.

A technical challenge was that the value of the monitoring responsivity could be changed with various bonding materials (of different refractive indexes), which was attributed to differences in the resultant reflectance at the sapphire/bonding material interface. To solve this problem, we deposited a layer of aluminum film on the backside of the sapphire substrate to serve as a reflector, so the reflectance from the sapphire bottom became independent of the bonding material or bonding quality. Moreover, the extraction efficiency of UV LEDs could be improved. The theoretical reflectance of the sapphire/aluminum interface at normal incidence was approximately 87%. We compared the performance of the same device in air before and after depositing an Al reflector. LEDs emit spontaneous emission isotropically. Some of the downward-emitting light was thus reflected upward by the Al reflector, and escaped from the top surface when the incident angle was smaller than the critical angle of the GaN/indium-tin oxide (ITO)/air interface, which led to a higher extraction efficiency and output power. In the meantime, the sapphire patterns (i.e., periodically etched sapphire cones) at the GaN/sapphire interface scattered some of the reflected light back to the sapphire slab waveguide, resulting in an additional MPD current, as shown in Figure 6. Moreover, another portion of the downward-emitting light with an incident angle larger and smaller than the critical angles of the GaN/ITO/air interface (approximately 23.6°) and sapphire/air interface (approximately 34.4°), respectively, which previously escaped from the sapphire bottom, then became trapped and propagated towards MPD. Therefore, the enhancement ratio in the MPD current can be greater than that in LED light power. Figure 7 illustrates the ratio of after to before Al deposition, in terms of UV LED light power, MPD current at zero bias and monitoring responsivity. The light power and MPD responsivity were improved by approximately 40% and 100%, respectively. (When the LED current was less than 3 mA, both the light power and MPD current were fairly low, lacking accuracy, resulting in fluctuations in the after-to-before ratios.)

## 6. Conclusions

Constant optical power operation of an UV LED was demonstrated by using a portable, low-cost module and a smartphone. The module consisted of an Arduino Uno card, a digital-to-analog converter, an integrated UV LED and MPD, a transimpedance amplifier and a Bluetooth transceiver. We wrote an Android App on a smartphone that remotely controlled the UV LED module via Bluetooth. The Arduino Uno card was programmed to receive demands from the smartphone, sent a driving voltage to the LED and returned the measured MPD voltage to the smartphone. A feedback loop was used to adjust the LED driving voltage to maintain a constant light output. No optical alignment was required due to the monolithic integration of the MPD. Constant optical power operation of UV LEDs would provide higher-quality assurance for disinfection or sterilization than constant current operation, though the life of UV LEDs could be shortened. The monitoring data can be shared through the smartphone and internet in real time, offering flexibility and quality control capability. Furthermore, when an Al reflector was deposited on the backside of the sapphire substrate, the LED’s light power and the MPD’s monitoring responsivity were enhanced by approximately 40% and 100%, respectively.

## Figures and Tables

**Table 1:** The key technical specifications of Arduino Uno [21].

Parameters	Value	Unit
Operating Voltage	5	V
Input Voltage (recommended)	7–12	V
Input Voltage (limit)	6–20	V
Digital I/O Pins	14	
Analog Input Pins	6	
DC Current per I/O Pin	20	mA
DC Current for 3.3 V Pin	50	mA
Flash Memory	32	KB
SRAM	2	KB
EEPROM	1	KB
Clock Speed	16	MHz
LED_BUILTIN	13	(Pin #)
Length × Width	68.6 × 53.4	mm^2^
Weight	25	g

**Table 2:** The key technical specifications of Bluetooth module HC-05 [22].

Parameters	Value	Unit
Operating Voltage	4–6	V
Operating Current	30	mA
Range	<100	m
Compatibility	Serial communication (USART) and TTL	
Protocol	IEEE 802.15.1	
Operating Mode	Master, Slave or Master/Slave	
Supported Baud Rate	9600, 19,200, 38,400, 57,600, 115,200, 230,400, 460,800	
